# Reproductive Pathological Changes Associated with Experimental Subchronic* Corynebacterium pseudotuberculosis* Infection in Nonpregnant Boer Does

**DOI:** 10.1155/2016/4624509

**Published:** 2016-02-23

**Authors:** A. M. Othman, Y. Abba, F. F. A. Jesse, Y. M. Ilyasu, A. A. Saharee, A. W. Haron, M. Zamri-Saad, M. A. M. Lila

**Affiliations:** ^1^Department of Veterinary Clinical Studies, Faculty of Veterinary Medicine, Universiti Putra Malaysia, 43400 Serdang, Selangor, Malaysia; ^2^Department of Pathology and Microbiology, Faculty of Veterinary Medicine, Universiti Putra Malaysia, 43400 Serdang, Selangor, Malaysia; ^3^Department of Veterinary Pathology, Faculty of Veterinary Medicine, University of Maiduguri, PMB 1069, Maiduguri 600233, Borno State, Nigeria; ^4^Research Centre for Ruminant Disease, Faculty of Veterinary Medicine, Universiti Putra Malaysia, 43400 Serdang, Selangor, Malaysia

## Abstract

*Corynebacterium pseudotuberculosis *causes caseous lymphadenitis (CLA), which is a contagious and chronic disease in sheep and goats. In order to assess the histopathological changes observed in the reproductive organs of nonpregnant does infected with the bacteria, 20 apparently healthy adult Boer does were divided into four inoculation groups, intradermal, intranasal, oral, and control, consisting of five goats each. Excluding the control group, which was unexposed, other does were inoculated with 10^7^ CFU/1 mL of live* C. pseudotuberculosis* through the various routes stated above. Thirty days after infection, the ovaries, uterus, and iliac lymph nodes were collected for bacterial recovery and molecular detection, as well as histopathological examination. The mean changes in necrosis, congestion, inflammatory cell infiltration, and oedema varied in severity among the ovaries, uterus, and iliac lymph nodes following different inoculation routes. Overall, the intranasal route of inoculation showed more severe (*p* < 0.05) lesions in all the organs examined. The findings of this study have shown that* C. pseudotuberculosis* could predispose to infertility resulting from pathological lesions in the uterus and ovaries of does.

## 1. Introduction

*Corynebacterium pseudotuberculosis* is gram-positive, facultative, anaerobic, small curved bacillus [1, 2]. It is generally regarded as an important animal pathogen causing caseous lymphadenitis (CLA) in sheep and goats. Several publications have reported that CLA can be transmitted through the oral, intradermal, intranasal, and intraperitoneal routes [3–7]. The organism has also been reported to cause significant economic losses to farmers due to hide and meat condemnation [8–11]. Recently, our study showed an increase in progesterone and estrogen levels in nonpregnant does inoculated with the bacterium; this may predispose to infertility since altered hormonal levels impair ovulation and implantation [7]. Based on the above study, there is a possibility that the bacteria might affect the reproductive organs of the doe and lead to pathological changes.

The pathogenesis of* C. pseudotuberculosis* in the reproductive organs is complex and not well understood even though it is believed that the bacteria disseminates through the afferent lymphatic system to the lymph node and internal organs, before multiplying within the macrophages as it survives the action of phagolysosomal enzymes [12, 13]. However, to better understand the mechanism of its pathogenesis with associated infertility, it is necessary to investigate the pathological changes in the reproductive organs and associated lymph nodes of hosts inoculated with the bacterium. Therefore, the present study aimed to investigate the histopathological changes in the reproductive organs and associated lymph nodes of nonpregnant does which were experimentally infected with* C. pseudotuberculosis* via intradermal, intranasal, and oral routes.

## 2. Materials and Method

### 2.1. Ethical Consideration

The experiment was conducted according to the guidelines of the international animal care and use committee, Universiti Putra Malaysia. The experimental procedure was conducted under the approval of the Animal Care and Use Ethics Committee (UPM/IACUC/AUP-R29/2014), Universiti Putra Malaysia, as required in Malaysia by the Animal Welfare Act (2014).

### 2.2. Animals and Management

Twenty (20) apparently healthy, nonpregnant Boer does with no history of CLA were used in this study. The goats were kept in separate pens. The temperature and ventilation of the pens were not regulated and goats were fed with commercial goat pellets (300 g/goat/day). Cut Napier grass, mineral block, and drinking water were given* ad libitum*. The does were acclimatized for two weeks, Blood samples and swab samples (oral, vaginal, and nasal) were collected for screening of CLA through bacterial isolation and PCR detection as described by Çetinkaya et al. [14]. Rectal temperature was monitored daily (morning and evening) and pregnancy examination was conducted one week before the experiment began using an ultrasound machine (brand-SIUI CTS 900V and probe 5.0 MHZ).

### 2.3. Estrus Synchronization

In order to avoid variations in cyclic changes, all the does were attempted to estrus synchronization. Intravaginal sponge containing 30 mg flurogesterone acetate (FGA) was inserted for 9 days. Forty-eight hours before sponge removal, cloprostenol (50 *μ*g) and pregnant mare serum gonadotropin (PMSG; 750 IU) were injected intramuscularly [15].

### 2.4. Bacterial Source and Reconfirmation

*Corynebacterium pseudotuberculosis* used in this study was obtained from a previous outbreak of caseous lymphadenitis in Taman Pertanian Universiti, Universiti Putra Malaysia [16]. The isolates were revived in a brain-heart infusion broth. The bacteria were recultured in 10% sheep blood agar and MacConkey's agar plates and incubated at 37°C for 48 hours. Furthermore, biochemical tests (catalase test, nitrate reduction test, urease, and fermentation of the sugars (glucose, sucrose, maltose, and xylose)) were performed for further confirmation of the bacterium.

### 2.5. Inoculum Preparation and Colony Forming Unit Counts

Few colonies from the blood agar plate were inoculated into a brain-heart infusion broth (BHI) and incubated in an incubator shaker set at 37°C, 150 rpm for 48 hours. The BHI broth was 10-fold serially diluted. One milliliter of 10^7^ dilution was used as inoculum and simultaneously plated onto blood agar plate for colony forming unit (CFU) count described by Alcamo [17].

### 2.6. Experimental Design

The 20 goats were divided randomly into four (Groups 1, 2, 3, and 4) equal groups. Groups 2, 3, and 4 were inoculated with 10^7^ cfu/1 mL of* C. pseudotuberculosis*, intradermally, intranasally, and orally, respectively. Group 1 was kept unexposed as a control group and was given 1 mL of sterile phosphate buffered saline (PBS) orally. All animals were observed for clinical signs daily and rectal temperature was monitored throughout the study period. Thirty days after infection, all animals were culled by exsanguination and postmortem examination was carried out to harvest the reproductive organs and their associated lymph nodes for bacterial isolation and identification of* C. pseudotuberculosis* and subsequent PCR detection.

### 2.7. Isolation and Detection of* C. pseudotuberculosis* from Tissues

Tissue samples from the reproductive organs and iliac lymph nodes were homogenized aseptically and cultured on blood agar and incubated for 48 hours at 37°C. Colonies with striking morphological characteristics (i.e., small, white, dry, and crumbly colonies) were subcultured onto new blood agar to get pure colonies. Biochemical tests (catalase test, nitrate reduction test, urease, and fermentation of the sugars (glucose, sucrose, maltose, and xylose)) were performed to confirm the bacterium.

### 2.8. DNA Extraction and Polymerase Chain Reaction (PCR) Confirmation

DNA was extracted from the bacterial cultures using DNAzol® according to the manufacturer's instructions (https://tools.thermofisher.com/content/sfs/manuals/10503.pdf). The PCR primers and master mix were purchased from IDNA Biotechnology Pte Ltd. The PCR was performed using a SensiQuest Thermocycler machine and was set for 30 cycles of amplification, following an initial denaturation step at 94°C for 5 minutes. Each cycle involved denaturation at 94°C for 1 minute, annealing at 56°C for 1 minute, synthesis at 72°C for 2 minutes, and final synthesis at 72°C for 2 minutes. For each PCR reaction tube, a total of 50 *μ*L reaction volume containing 2 *μ*L DNA as template, 2 *μ*L of 25 Mm MgCl_2_, 2 *μ*L of 10x Taq buffer with (NH_4_)_2_SO_4_, 0.5 *μ*L of 10 mM dNTP mix, 0.5 *μ*L forward primer, 0.5 *μ*L reverse primer, 0.5 *μ*L Taq DNA polymerase (5 *μ*/*μ*L), and 42 *μ*L sterile distilled water were added, respectively. The amplified products were analyzed by electrophoresis on 1% (w/v) agarose gel with the addition of 1.5 *μ*L FloroSafe DNA stain and run at 60 volts (V), 350 milliampere (mA) for 70 minutes. The oligonucleotide primer used in this study was 16S rRNA gene [14]. The forward primer used was (5′-CCGCACTTTAGTGTGTGTG-3′) and the reversed primer was (5′-TCTCTACGCCGATCTTGTAT-3′), respectively. The PCR products were detected as* C. pseudotuberculosis* according to molecular size of 816 bp as documented by Çetinkaya et al. [14].

### 2.9. Histopathology

The isolated reproductive organs (ovary and uterus) and the associated lymph node (iliac lymph node) were collected and fixed at 10% buffered formalin for 3 days before processing. The tissues were processed in an automatic tissue processor (Leica TP 1020, Germany) and then embedded in paraffin blocks, sectioned at 5 *μ* and stained with standard hematoxylin and eosin (H&E) stain for light microscopic evaluation [18].

### 2.10. Histological Lesion Scoring

Based on the methods described by Henry et al. [19] and Hair-Bejo et al. [20], the histopathological scoring was performed following observation of ten slides from each organ. About six microscopic areas for each slide were observed at different magnification (200x and 400x). The lesion of the three inoculated groups and control was scored on a scale of 0–3 based on the presence of oedema, necrosis, congestion, infiltration of neutrophils, degeneration, and necrosis. Score 0 indicates normal (no lesion observed), 1 mild (less than 30% lesion observed), 2 moderate (less than 60% of the lesion observed), and 3 severe (more than 60% of the lesion observed).

### 2.11. Statistical Analysis

Data were analyzed using statistical software Graph Pad Prism (version 6.0). One way ANOVA with Kruskal-Wallis nonparametric test was used to test the differences between the lesion scores in the different inoculation groups. The differences were considered as significant at *p* < 0.05.

## 3. Results

### 3.1. Bacterial Reconfirmation

The biochemical test carried out confirmed the bacteria as* C. pseudotuberculosis*. The results were catalase positive, nitrate reduction, and urease positive.

### 3.2. PCR Confirmation and Analysis

The 816 bp segment of the 16S RNA is unique to* C. pseudotuberculosis* and was detected by PCR. Bacterial isolations from tissues of goats (ovary, uterus, and iliac lymph nodes) inoculated through the intranasal and oral inoculation routes tested positive for the 16S RNA, while goats' inoculated intradermally tested PCR positive only from bacterial cultures of the iliac lymph node.

### 3.3. Histopathological Changes

**Figure 1:**
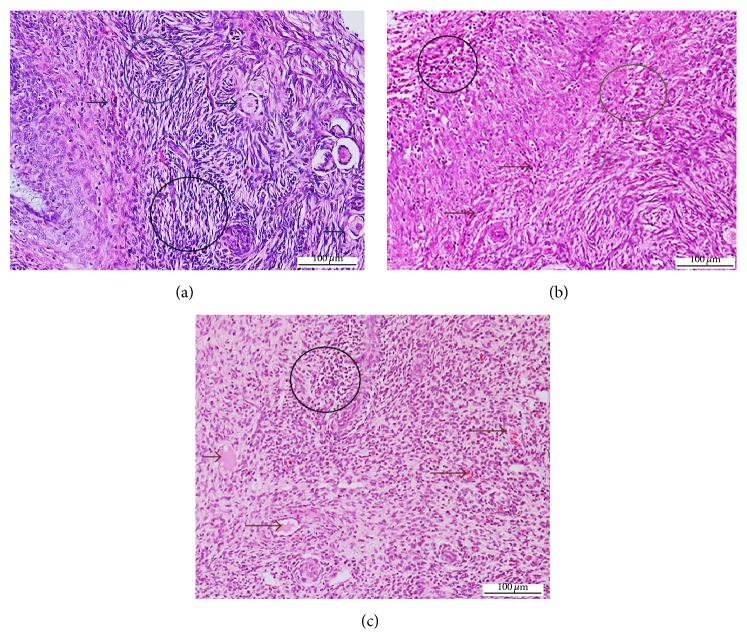
Photomicrograph section of the ovary of nonpregnant does inoculated with* Corynebacterium pseudotuberculosis*: (a) inflammatory and necrotic cells (circle) following intranasal inoculation; (b) inflammatory and necrotic cells (circle), congestion (brown arrows), following oral inoculation; (c) congestion (black arrows), inflammatory and necrotic cells (black circle), following intradermal inoculation (H&E ×200).

The mean changes in necrosis and congestion in the ovaries were moderate to severe in all groups and were not significant (*p* > 0.05) between the three inoculation groups (Table 1). However, inflammatory cell infiltration in the ovary was mild in the intradermal group and moderate to severe in both oral and intranasal groups (Figures 1(a), 1(b), and 1(c)).

**Figure 2:**
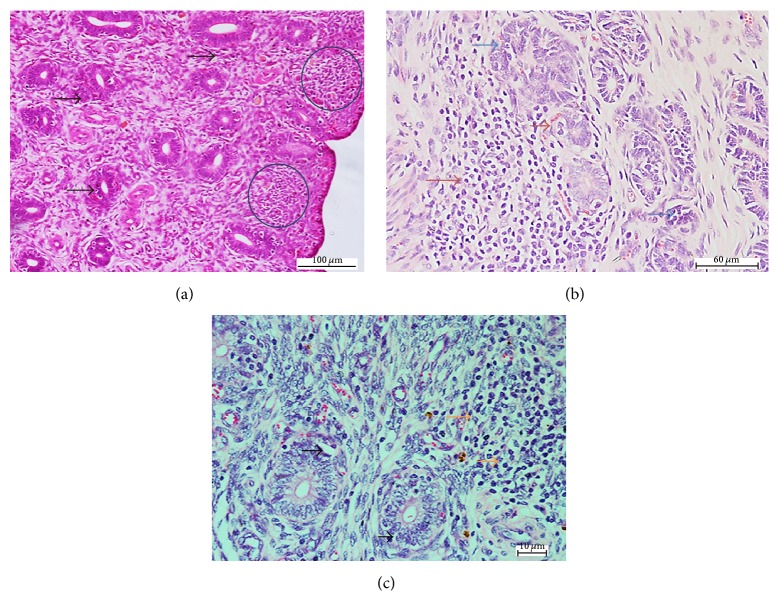
Photomicrograph section of the uterus of nonpregnant does inoculated with* Corynebacterium pseudotuberculosis.* Note (a) the inflammatory and necrotic cells (brown arrows), degeneration of endometrial glandular epithelium (blue arrows), following intranasal inoculation; (b) degeneration of endometrial glandular cells (black arrows), inflammatory cells (orange arrow), following oral inoculation; (c) inflammatory cells in the submucosa (blue circle), degeneration and necrosis of endometrial glandular epithelium (black arrow), following intradermal inoculation (H&E ×200).

The mean changes in necrosis, inflammatory cell, and congestion in the uterus were mild to moderate following intradermal and oral inoculation and moderate to severe following intranasal inoculation (Table 2). These changes were significantly higher (*p* < 0.05) in the intranasal group and comparable in the intradermal and oral groups. On the other hand, edema was comparable in the uterus following intranasal and oral inoculation (*p* > 0.05) and significantly lower (*p* < 0.05) in the intradermal group (Figures 2(a), 2(b), and 2(c)).

**Figure 3:**
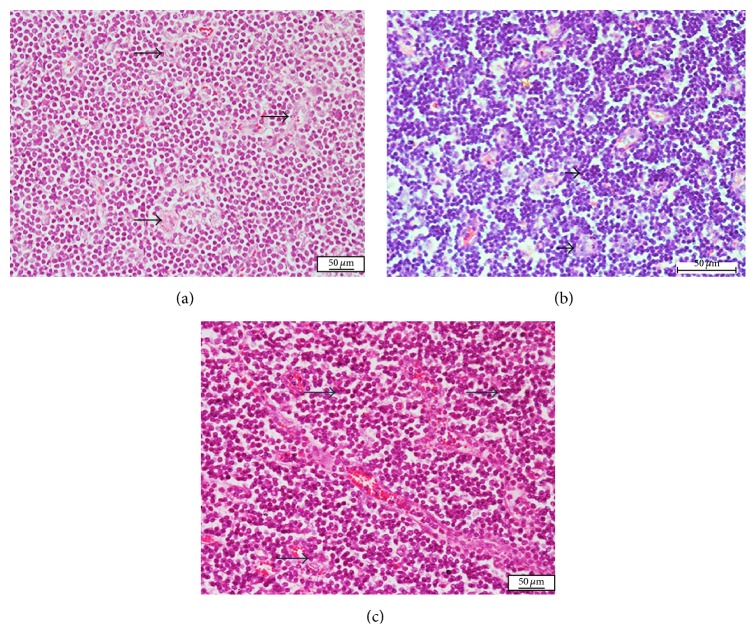
Photomicrograph section of the lymph node of nonpregnant does inoculated with* Corynebacterium pseudotuberculosis*: Note (a) the neutrophil infiltration and microabscesses with a central necrotic areas (black and orange arrows), following intranasal inoculation; (b) inflammatory cell infiltration characterized by neutrophils and macrophages (black arrows), congestion (blue arrow), following oral inoculation; (c) neutrophil infiltration and microabscesses with central necrotic areas (black arrows), following intradermal inoculation (H&E ×400).

In the iliac lymph nodes, distribution of abscess was moderate to severe and comparable (*p* > 0.05) in the intradermal and oral groups, while it was severe in the intranasal group (Table 3). However, distribution of inflammatory cells and congestion is mild to moderate in the intradermal group, while it was severe and comparable in both intranasal and oral inoculation groups (Figures 3(a), 3(b), and 3(c)).

## 4. Discussion

The present study reports, for the first time, the changes observed in the reproductive organs and associated lymph nodes in nonpregnant does experimentally infected with* C. pseudotuberculosis* via intradermal, intranasal, and oral routes. All animals inoculated with the organism through different routes developed significant histopathological changes in the reproductive organs compared to the control groups; however, goats inoculated through the intranasal route showed severe lesion in the reproductive organs compared to other routes.

The histopathological lesions we observed in the ovary were comparable with those observed in the uterine horn. These lesions may have been induced by either the bacteria or its phospholipase D and might explain the hormonal alterations observed in our earlier study [7]. All the lesions observed in the ovary in the present study were similar to those described by Khuder et al. [21] in female mice experimentally inoculated with* C. pseudotuberculosis* and its phospholipase D (PLD). The authors observed infiltration of leukocytes in the lumen of ovulated follicles and generalized congestion, thrombosis, degeneration, and necrosis of the stromal cells of the ovary. Changes observed in the ovaries of all does inoculated through the different routes can be associated with severe biological activity such as dermonecrosis, complete capillary destruction caused by the bacterial exotoxin [22–24]. However, this explains the presence of necrosis and congestion of the blood vessel in the present study. Edema found in the uterus of all goats after inoculation may also be due to the presence of the exotoxin, phospholipase D (PLD). Carne and Onon [25] stated that hydrolysis of sphingomyelin and leakage of plasma protein into surrounding tissues may occur as a result of increase in vascular endothelial membrane permeability. In our previous study [7], we investigated the changes in reproductive hormonal levels of nonpregnant does inoculated with live* C. pseudotuberculosis* and reported increased levels of progesterone and estrogen thirty days after infection in all inoculation groups. These hormonal changes could be attributed to the pathological conditions we observed in the ovary in this study. The abnormal increase of progesterone may also be translated as a sign of pseudo pregnancy, thus impairing normal follicular development and ovulation. Foster [26] stated that stated that pseudo pregnancy in nonpregnant animals may result from mucometra or hydrometra in the uterus, which may lead to increase in progesterone secretion. Microabscesses were observed in the iliac lymph nodes and are considered among the typical signs of the disease. These findings are supported by Adza Rina et al. [6], who found that goats inoculated with 10^7^ concentrations of live* C. pseudotuberculosis* through intranasal route developed abscesses in the mesenteric and supramammary lymph nodes compared to oral and intradermal routes. Similarly, Mahmood et al. [27] observed severe abscess formation, congestion, hemorrhage, degeneration and necrosis, and inflammatory cellular infiltration in Boer goats subcutaneously inoculated with* C. pseudotuberculosis* as compared to those inoculated intravenously with its phospholipase D. However, all of these studies failed to investigate the association of the iliac lymph nodes and pathological changes in the female reproductive organs such as the uterus and ovaries.

Bacterial isolation showed presence of the organism in all the tissues following oral and intranasal inoculation, but only in the lymph node following intradermal inoculation. Since macrophages and dendritic cells are the first to come in contact with an organism following inoculation through the intradermal route, these phagocytes likely engulfed the bacteria and were carried to the lymph nodes through the lymphatic drainage and the bacteria eventually multiplied in the nodes in the other tissues. Valli and Parry [28] reported that CLA lesions are normally present in the internal organs, udder, and less common in the uterus, testis, and scrotum. In a related study, Junior et al. [29] reported the development of mastitis and increased systemic neutrophilia following intradermal inoculation of* C. pseudotuberculosis* in the mammary gland of goats. The researchers also observed enlarged supramammary lymph nodes without abscess formation.

## 5. Conclusion

This study showed that the intranasal route was the most effective route for CLA infection of the reproductive system as evidenced by pathological lesions observed in these tissues. This study further corroborates with our previous findings that CLA infection in nonpregnant does may lead to infertility resulting from pathological changes in the reproductive organs as well as an imbalance in reproductive hormonal levels.

## Figures and Tables

**Table 1:** Mean scores of histopathological changes in the ovaries of nonpregnant Boer does experimentally inoculated with *C. pseudotuberculosis*.

Parameters	Inoculation groups			
	Control	Intradermal	Intranasal	Oral
Necrosis	0.0 ± 0.00^a^	2.40 ± 0.69^b^	2.60 ± 0.52^b^	2.40 ± 0.52^b^
Inflammatory cells	0.0 ± 0.00^a^	1.0 ± 0.00^b^	2.90 ± 0.32^c^	2.50 ± 0.53^c^
Congestion	0.0 ± 0.00^a^	1.80 ± 0.42^b^	2.40 ± 0.52^b^	1.50 ± 0.53^c^

^a,b,c^Means with different superscripts within the same row are significantly different from each other at *p* < 0.05.

**Table 2:** Mean scores of histopathological changes in uterus of nonpregnant Boer does experimentally inoculated with *C. pseudotuberculosis*.

Parameters	Group			
	Control	Intradermal	Intranasal	Oral
Necrosis	0.0 ± 0.00^a^	0.30 ± 0.48^a^	3.00 ± 0.00^b^	1.00 ± 0.00^c^
Inflammatory cell	0.0 ± 0.00^a^	1.0 ± 0.00^b^	2.50 ± 0.53^c^	1.50 ± 0.53^b^
Congestion	0.0 ± 0.00^a^	1.20 ± 0.92^b^	3.00 ± 0.00^c^	2.00 ± 0.00^b^
Edema	0.0 ± 0.00^a^	0.80 ± 0.42^b^	2.80 ± 0.42^c^	2.60 ± 0.69^c^

^a,b,c^Means with different superscripts within the same row are significantly different from each other at *p* < 0.05.

**Table 3:** Mean scores of pathological changes in lymph node of nonpregnant Boer does experimentally inoculated with *C. pseudotuberculosis*.

Parameters	Group			
	Control	Intradermal	Intranasal	Oral
Abscess	0.00 ± 0.0^a^	2.20 ± 0.42^b^	3.00 ± 0.0^c^	2.30 ± 0.48^b^
Inflammatory cells	0.00 ± 0.0^a^	1.60 ± 0.52^b^	3.00 ± 0.0^c^	3.00 ± 0.00^c^
Congestion	0.00 ± 0.0^a^	1.60 ± 0.52^b^	3.00 ± 0.0^c^	3.00 ± 0.0^c^

^a,b,c^Means with different superscripts within the same row are significantly different from each other at *p* < 0.05.
